# Barriers and Facilitators of Using Sensored Medication Adherence Devices in a Diverse Sample of Patients With Multiple Myeloma: Qualitative Study

**DOI:** 10.2196/cancer.9918

**Published:** 2018-11-12

**Authors:** Alemseged Ayele Asfaw, Connie H Yan, Karen Sweiss, Scott Wirth, Victor H Ramirez, Pritesh R Patel, Lisa K Sharp

**Affiliations:** 1 Pharmacy Systems, Outcomes & Policy College of Pharmacy University of Illinois at Chicago Chicago, IL United States; 2 Pharmacy Practice College of Pharmacy University of Illinois at Chicago Chicago, IL United States; 3 College of Medicine University of Illinois at Chicago Chicago, IL United States; 4 Department of Medicine, Section of Hematology/Oncology College of Medicine University of Illinois at Chicago Chicago, IL United States

**Keywords:** antineoplastic therapy, challenges, race/ethnicity, medication adherence, multiple myeloma

## Abstract

**Background:**

Many recently approved medications to manage multiple myeloma (MM) are oral, require supportive medications to prevent adverse effects, and are taken under complex schedules. Medication adherence is a concern; however, little attention has been directed toward understanding adherence in MM or associated barriers and facilitators. Advanced sensored medication devices (SMDs) offer opportunities to intervene; however, acceptability among patients with MM, particularly African American patients, is untested.

**Objective:**

This study aimed to explore patients’ (1) perceptions of their health before MM including experiences with chronic medications, (2) perceptions of adherence barriers and facilitators, and (3) attitudes toward using SMDs.

**Methods:**

An in-person, semistructured, qualitative interview was conducted with a convenience sample of patients being treated for MM. Patients were recruited from within an urban, minority-serving, academic medical center that had an established cancer center. A standardized interview guide included questions targeting medication use, attitudes, adherence, barriers, and facilitators. Demographics included the use of cell phone technology. Patients were shown 2 different pill bottles with sensor technology—Medication Event Monitoring System and the SMRxT bottle. After receiving information on the transmission ability of the bottles, patients were asked to discuss their reactions and concerns with the idea of using such a device. Medical records were reviewed to capture information on medication and diagnoses. The interviews were audio-recorded and transcribed. Interviews were independently coded by 2 members of the team with a third member providing guidance.

**Results:**

A total of 20 patients with a mean age of 56 years (median=59 years; range=29-71 years) participated in this study and 80% (16/20) were African American. In addition, 18 (90%, 18/20) owned a smartphone and 85% (17/20) were comfortable using the internet, text messaging, and cell phone apps. The average number of medications reported per patient was 13 medications (median=10; range=3-24). Moreover, 14 (70%, 14/20) patients reported missed doses for a range of reasons such as fatigue, feeling ill, a busy schedule, forgetting, or side effects. Interest in using an SMD ranged from great interest to complete lack of interest. Examples of concerns related to the SMDs included privacy issues, potential added cost, and the size of the bottle (ie, too large). Despite the concerns, 60% (12/20) of the patients expressed interest in trying a bottle in the future.

**Conclusions:**

Results identified numerous patient-reported barriers and facilitators to missed doses of oral anticancer therapy. Many appear to be potentially mutable if uncovered and addressed. SMDs may allow for capture of these data. Although patients expressed concerns with SMDs, most remained willing to use one. A feasibility trial with SMDs is planned.

## Introduction

### Background

Cancer treatment is being transformed by the rapid expansion of novel oral therapies [1,2]. While greater than 50 oral anticancer (OAC) medications are currently approved and in use, many more are in development [1,3]. The transition to oral routes of administration offers potential benefits to patients and providers, but new challenges are also introduced. This new paradigm places significant responsibility on patients to manage their medications autonomously outside of the clinical setting. Moreover, 2 literature reviews found adherence rates to OAC medications across all cancer types ranged from 40% to 100% including 20% to 44% of patients who took more medication than directed [4,5]. These results are of concern because taking OAC medication in amounts other than the directed dosage can significantly reduce the efficacy of OAC therapies while contributing to adverse events and economic waste [6].

Multiple myeloma (MM) is a specific example of cancer where novel oral therapies have resulted in vast improvements in survival over the past decade [7,8]. We could find no empirical data exclusively addressing medication adherence in patients undergoing treatment for MM; however, concern is warranted. Risk factors for poor adherence to OAC medications include the number of medications prescribed, being older in age, associated side effects, costs associated with treatment, and identifying as an ethnic minority among others [5]. These factors are relevant for many patients with MM. The treatment regimen for MM is among the more complex of cancer treatments relying on multiple oral medications that need to be taken on irregular schedules. Patients are typically treated with 1 of the several OAC medications such as thalidomide [9], lenalidomide [10], pomalidomide [11], ixazomib [12], and panobinostat [13] combined with oral steroids. In addition, supportive care focuses on prevention of infectious complications, pulmonary emboli, and bone morbidity, which involves additional medications contributing to the overall medication burden [14]. The cost of OAC therapies for MM can exceed US $20,000 per month, and insurance coverage varies tremendously [15,16]. Most MM cases are diagnosed in the elderly aged 65 years and above [17] who present with pre-existing age-related chronic health problems that require daily medications to manage. Finally, for unknown reasons, African Americans are at a higher risk of being diagnosed with MM than other racial and ethnic groups.

A fundamental limitation in the field of medication adherence is measurement of real-time medication-taking behavior. To date, few interventions have been conducted to improve adherence to OAC medications, and results suggest that additional research is needed to further refine intervention development [18]. Ethnic minorities and individuals from lower socioeconomic backgrounds may be particularly vulnerable to adherence challenges [19]. With this in mind, the study team was interested in exploring the potential of using technology to capture patients’ medication-taking behaviors as 1 component of a future intervention. Numerous real-time or sensored medication devices (SMDs) are currently available with additional devices under development [20]. The specific operations of each SMD vary widely. Simple SMDs include specialized caps or lids that fit onto traditional medication bottles and provide an alarm feature (ie sound or light) that can be scheduled at specified times based on medication regimen. Some have the added feature of recording the date and time of cap removal. More sophisticated SMDs provide the auditory and visual alarm in addition to transmitting *real-time* information to patients via text messages as well as texts or telephone calls to caregivers or providers. These are often supported by internet-based apps that track the date and time of device openings in visual graphics.

From an intervention development perspective, the more advanced SMDs are appealing because they transmit real-time information on missed device openings. Specially, when alerted that a patient missed a dose, researchers may be able to communicate with the patient to understand the contextual factors associated with each missed dose as it occurs. In turn, this information may allow for tailored medication support interventions that are more accurately matched to each patient’s unique barriers. However, a fundamental question must be answered before intervention development is initiated—are SMDs that track patients’ behavior and transmit real-time data acceptable to patients? Inclusion of African Americans was critical, considering that their rates of MM are higher [21].

### Objective

This study was undertaken with the long-term goal of developing a patient-centered intervention to support adherence to OAC medications. Patients with MM were targeted because no data on adherence to MM regimens were identified despite numerous factors placing them at high risk for poor adherence. This initial phase of research aimed to understand patients’ (1) perceptions of their health before MM including experiences with chronic medications, (2) perceptions of adherence barriers and facilitators, and (3) attitudes toward using SMDs. Although not a primary aim, information was collected on cell phone ownership, use of cell phone functions, and the internet to understand the degree to which the sample was comfortable with basic technology that might be integrated into an intervention.

## Methods

### Participants and Recruitment

A convenience sample of patients was recruited from within an urban, minority-serving, academic medical center with an established cancer center. Patients were recruited as they presented for a regularly scheduled appointment with their established oncology team. Eligibility criteria were as follows: (1) aged 18 years or older, (2) current diagnosis of MM, (3) receiving orally administered oncology treatment for MM currently or in the past 3 months, and (4) English speaker. Before the days on which the MM clinic visit was scheduled, the research assistant spoke with the oncologist and 2 oncology pharmacists to identify scheduled patients who were qualified. When the patient presented to the clinic, a provider assessed interest in the research. Willing patients met the interviewers in a private conference room to sign the informed consent form and conduct the interview. Interviews were audio-recorded.

### Development of the Interview Guide

A semistructured interview guide was drafted by a health psychologist with formal training in qualitative research (LKS) and experience working with diverse populations on adherence. An oncology physician and 2 pharmacists reviewed the interview draft of 12 open-ended questions for accuracy, clarity, and content from a medical perspective. To address the patients’ perspective, the revised 9-item interview guide was assessed in 1 MM patient who responded to the questions and provided feedback. A final interview guide consisted of 9 open-ended questions (shown in Multimedia Appendix 1). Full ethics review and approval was obtained from the institutional review board.

### Data Collection

All interviews were conducted by 2 trained team members, (LKS, VR) that began by assessing the type of cell phone ownership and use of short message service texts, internet, or internet apps. Although this was not a stated aim, experience with these technologies could influence responses to the interview and had implications for intervention design. As shown in Multimedia Appendix 1, additional questions addressed (1) the patient’s health before being diagnosed with MM including pre-existing chronic comorbidities and experience with daily medications, (2) current health and medications, (3) priority of medications, (4) organization of medications, (5) perceived barriers and facilitators to adherence, and (6) perspectives on 2 specific SMDs. Before asking about perspectives on SMDs, all patients were allowed to hold and manipulate 2 different pill bottles with sensor technology. They did not have the opportunity to actually use the bottles themselves. The 2 devices used in this study were MEMS (Medication Event Monitoring System) bottle and the SMRxT bottle. These were selected because they were accessible to our team. The interviewers provided information on the transmission ability of the bottles. Of note, the MEMS bottle recorded information that was downloaded later, whereas the SMRxT bottle transmitted information in real time. Demographic information and number of prescribed medications were abstracted from the electronic medical records to lessen patient burden. Patients were provided US $20 reimbursement for their time.

### Data Analysis

Audio-recorded interviews were transcribed as they were completed. The lead investigator determined that thematic saturation had been attained after 20 interviews conducted over 2 months yielded no new information. In addition, the demographic characteristics of respondents were evaluated to ensure that premature saturation was not met due to homogeneous sample selection.

Coding followed a specific type of thematic analysis known as the framework method [22]. This deductive approach is often used in health-related research designed to answer specific questions with qualitative inquiry. Like all thematic analysis, the framework method involves developing an analytic framework of codes that is applied by independent coding of transcripts. To achieve this coding framework, 3 members of the research team (AAA, CHY, and LKS) with training in qualitative research independently read the transcripts and met twice to establish the application of the initial codes. Subsequently, the 3 members independently coded 2 randomly selected transcripts and met again to compare codes. Following discussion and minor modifications to the coding framework, the 3 members independently coded the remaining transcripts, managing the data in Microsoft Excel. The 3 members met to compare results and reached consensus on coding for all 20 transcripts.

## Results

### Sociodemographic Characteristics

A total of 20 patients were approached for participation and all agreed, resulting in a 100% response rate. The length of the interview varied from 20 to 40 min. As shown in Table 1, the median age of patients was 59 years (range 29-71 years) and 80% (16/20) were African American. The median time since being diagnosed with MM was 25.5 months, with a range from 2 months to 192 months. Consistent with the demographics of the larger patient population seen in this setting, the majority of participants were covered by government-issued insurance (Medicaid or Medicare). Although 7 patients had private insurance, 3 of these individuals expressed concern regarding the future of their coverage. Moreover, 2 patients were concerned that the insurance might “run out,” and 1 male who had to stop working because of the MM stated that his insurance was only covering him for a few more months.

**Table 1:** Sociodemographic characteristics of study participants.

Characteristics		Statistics
**Sex, n (%)**		
	Female	9 (45)
	Male	11 (55)
**Age in years**		
	Median	59
	Range	29-71
**Race/ethnicity, n (%)**		
	African American	16 (80)
	White	3 (15)
	Hispanic/Latino	1 (5)
**Marital status, n (%)**		
	Married	10 (50)
	Single	8 (40)
	Widow	1 (5)
	Divorced	1 (5)
**Type of insurance, n (%)**		
	Private	7 (35)
	Medicaid	6 (30)
	Medicare B	6 (30)
	Uninsured	1 (5)

### Cell Phone Ownership and Use of Internet

Out of the 18 (90%, 18/20) participants who reported owning an internet-enabled cell phone, 17 used it to navigate the internet, interact with apps, and text message. One male aged 60 years who owned a smartphone stated that he did not like to type so he did not use text messaging or cell phone apps. However, he did use his phone to access the internet, which required typing, and he also took pictures with his phone. The 2 female participants with basic phones that were not internet-enabled were aged 63 and 67 years, respectively. One owned a government-subsidized phone and did not know how to use the internet, phone apps, or text messaging. The second woman stated that she used her flip phone for emergencies only and rarely switched it on. She did not know how to use text messaging. Instead of a cell phone or texting, she preferred to use email on her computer, which she did frequently. She did not know how to use the internet on her phone. Importantly, this participant also had multiple sclerosis that affected her ability to use her hands at times. As a result, the keyboard on a computer was easier for her to use than a cell phone.

### Experiences With Pre-Existing Disease and Current Number of Medications

A total of 10 individuals described feeling healthy and free of any chronic health problems requiring daily medications before their diagnosis of MM. Prior health issues consisted of minor injuries, colds, or broken bones. Not surprisingly, they recalled feeling overwhelmed or confused when they initiated their treatments for MM; however, that dissipated over time as they became more accustomed to the routine.

The remaining 10 patients reported having a range of pre-existing chronic diseases at the time they were diagnosed with MM: hypertension (n=3), high cholesterol, diabetes, multiple sclerosis, anemia, a mental health problem, chronic obstructive pulmonary disease, and goiter. All these patients required chronic daily medication except for a single 40-year-old African American female whose goiter required monitoring but no daily medications. At the time of the interview, medical records revealed that all 20 patients had multiple chronic diseases requiring daily medications. The median number of chronic medications was 10, with a range of 3 to 24 per person including those for MM.

### Perspectives on Prioritizing Medications for Multiple Myeloma and Chronic Disease

Three distinct subthemes emerged within a larger medication priority theme: (1) the cancer medicine was the most important, (2) the cancer medicine and warfarin were the most important, or (3) all medicines were equally important. Furthermore, 2 patients expressed the feeling that their *cancer medication* was the most important among all their medications, referring specifically to the names of their respective OAC medication. Both conveyed the sense that their life depended on their adherence to the OAC medication. Interestingly, neither viewed the supportive medications used to manage their cancer- (ie, osteoporosis and anemia) and treatment-related effects as being a *cancer medication*. A total of 2 patients identified their OAC medication along with their *blood thinner* (ie, warfarin) as being the most important, despite being on additional medications for comorbid diseases. They both understood that the *blood thinner* prevented them from having blood clots, which was a serious side effect from some of the OAC medications. The remaining 16 patients expressed a sense that all of their medications, for both cancer and chronic conditions, were needed to improve their health.

However, there were nuanced variations in why individuals felt their chronic disease medications were important. A total of 4 patients explained that their past medical experiences such as a myocardial infarct, worsening symptoms of multiple sclerosis, or symptoms of mental illness led them to conclude that the medications for chronic diseases were equally important. For example, a 60-year-old married African American male with MM for 60 months stated:

I had a heart attack, so that has solidified and strengthened my belief that my hypertension medication is just as important as my cancer medication. If I don’t take it, I’m in danger of getting a heart attack again.

For others, the belief that specific adverse outcomes might occur if they were to stop their medications for chronic diseases was sufficient to motivate medication adherence. Most provided more general explanations for their beliefs, suggesting that all the medications worked together in some manner to maintain their health.

### Barriers to Oral Anticancer Medication Adherence and Missed Doses

Barriers to medication adherence were defined as factors that contributed to missed doses of MM medications. MM medications included OAC medications as well as the adjunct or supportive medications prescribed to minimize the adverse effects of the OAC medications. Despite expressing their feelings about the importance of medications, 14 (70%, 14/20) patients stated that they sometimes did miss doses of their OAC medications. Most of the patients described situations where the missed doses were unintentional. In addition, 10 major thematic areas related to barriers to medication adherence were identified in the interviews: side effects, distractions, insurance or pharmacy delays, number of medications, travel or being away from home, pill size, fatigue, stigma, homelessness, and spirituality.

#### Side Effects

A total of 6 (30%, 6/20) patients noted medication side effects as a barrier to taking their OAC medications as prescribed. Examples of side effects included stomach discomfort, diarrhea, vomiting, fatigue, stiff legs, ankle swelling, foot tingling, or constipation. Although 2 patients boldly explained that they intentionally skipped doses of their cancer medications to avoid side effects, others described approaches to decrease side effects such as taking the medications that they felt caused the side effects at night while they slept or making sure to eat food before taking the medication. Most of the patients expressed that although the side effects were bothersome, they were usually willing to tolerate them recognizing the benefit of the OAC medicine.

#### Distractions

A total of 6/20 (30%) patients described distractions of various types that contributed to missed or delayed OAC medication doses. Distractions were a broad theme that encompassed events such as celebrating birthdays or holidays, rushing or moving quickly in response to something, being busy, and becoming involved in an activity such as a hobby or watching television. A married African American male with MM for 60 months reported:

I don’t really forget to take my medicine completely [on weekends], I just don’t take it at the same time as during the week days.

The interviews implied that the patients’ normal routine was altered in some way or they lost track of time due to the situation.

#### Insurance or Pharmacy Barriers

A total of 6 (30%, 6/20) patients reported experiencing health care system barriers that made it difficult to obtain their OAC medications at some point in their treatment. Specifically, 5 attributed delays in accessing their medication to their insurance companies. One patient reported missing at least 1 dose of his OAC due to the delay and another had a delay in treatment initiation. In addition to insurance barriers, 1 patient described that he had difficulty getting OAC medication refills on time due to what he perceived to be miscommunications between the oncologist and the pharmacy. In his words, “I have to figure out where my medications are.” He described having to obtain an “emergency supply from a different pharmacy” to prevent missed doses.

#### Number of Medications

The number of medications patients were asked to take was mentioned negatively or as a burden by 6 (30%, 6/20) patients. Comments ranged from feelings of frustration or worry to being overwhelming or just feeling the number was excessive. Although some of the patients were experiencing the burden at the time of the interview, a few commented that their feelings of being overwhelmed by the medications was heightened earlier in their MM treatment but had subsided with a decrease in the number of medications prescribed at the time of the interview. A 41-year-old African American female with MM for 31 months who was on 24 medications stated:

I just worry that I am taking so many pills. Sometimes it’s psychological when I feel that my throat closes up - refuses to swallow them. They won’t go down. It’s like my body is rejecting them but I have to force it thru.

Pill burden was often given as a reason for forgetting to take medication.

#### Travel or Being Away From Home

A total of 3 (15%, 3/20) patients mentioned that when they were away from home or had a night out, they just took their evening medications whenever they returned home. As a result, they took their evening medications, including the OAC medication, at irregular intervals or skipped doses.

#### Pill Size

A total of 3 (15%, 3/20) patients mentioned the size of the calcium pill as being a barrier that often resulted in missed doses. In the context of MM, calcium is often prescribed as an adjunct to treatment aimed at supporting bone health [23].

#### Fatigue

Fatigue was a common side effect of the medications; however, 2 (10%, 2/20) patients mentioned fatigue in relation to adherence. They commented that their nighttime medication was the hardest to adhere to because of feeling tired at the end of the day. At times, they fell asleep without taking their medication, which resulted in either taking the medication off schedule when they awakened during the night or missing the dose entirely.

#### Stigma

A total of 2 (10%, 2/20) patients made comments that reflected a stigma associated with the need for medications; however, they were subtly different. The youngest participant, a 29-year-old married African American male with MM for 7 months, reported that he missed his medications when he was “getting high with his homies.” Although substance abuse could be considered the key barrier, a careful analysis of his transcript suggested otherwise. As this young man spontaneously explained, he kept all his medications in their bottles next to his bed, and he had considered just taking the bag with him when he partied with his friends. However, he had not told his friends that he had cancer, as he feared the stigma and rejection if they knew he was ill. The second example came from a 68-year-old married African American male who commented that all his medications “make my house look like a drug house,” explaining that his son had a drug problem. Although he reported rarely missing his medications, he strongly disapproved of medications in general and struggled with his own need for them.

#### Homelessness and Spirituality

A 60-year-old single African American woman with MM for 53 months was the only patient who did not describe any location for keeping her medications, which was attributed to the fact that she was homeless and resided in shelters at times. Despite the lack of any consistent location for her medications, she stated that she tried to take them in the morning if she ate breakfast. She did mention using a pillbox in the past, but she was not using one at the time of the interview ostensibly because she could not keep up with it. She was very open about not being adherent to her medications throughout the interview, which was a great concern to her oncologist who was aware of the situation. However, she expressed that she was “a strong believer in God”; therefore, she did not worry when she missed her medications.

#### Cost of Medications

Although all patients were able to financially access OAC medications at the time of the interview, financial concerns for the future were common and impacted life choices for some. For example, 2 patients reported concern that their private employer-based insurance was reaching the limit soon and they were not able to return to work. Neither were clear on how they would afford health care or medications once their insurance coverage ended. Conversely, a 40-year-old single African American female was interested in working but feared losing Medicaid coverage if she returned to work. This was complicated by the fact that historically she had not found positions that offered employer-based health insurance. One 65-year-old single African American male reported that he was currently receiving his OAC medication with assistance from a patient access network. However, in his words, he “did not know how long this lasts” and felt that when it ended, he would have to decide if he wanted to “become broke or die.” Most patients reported having manageable co-payments for their medications ranging from US $2 to US $15; although not everyone reported the exact cost. Several patients with insurance reported having no co-pay. The setting offered numerous medication financial assistance programs (ie, foundations and access networks) for patients who did not have insurance coverage.

### Facilitators to Medication Adherence

Facilitators to medication adherence were defined as factors that aided patients in adhering to MM medication. As with barriers, MM medications included OAC medications and the adjunct or supportive medications. A total of 5 major thematic areas were identified in the interviews: location of medicines, organization of medicines, medication reminders, social support, and spirituality.

#### Location of Medications

The most common theme, identified in all but 1 patient’s interview, was related to having a special location to keep medications. In describing their unique locations for storing medications, it was clear that most had a reason for the location selection. Several patients focused on selecting locations where they thought they would be when they needed to take the medications. For example, locating bottles on the top of a nightstand in the bedroom was strategic because “they [the bottles] are the first thing I see when I wake up and go to bed.” Other locations included the top of the bedroom dresser, on the kitchen counter, or kitchen table. The kitchen was popular for those who took medications around mealtimes. Only a 41-year-old widowed African American female with MM for 31 months who was on 24 different medications described multiple locations for storing her medications. She stored her medications together based on the health problem they targeted. For example, all her cancer medications were in a drawer and all her blood pressure medications were in a cabinet. Although less common, a few patients preferred to keep their medications out of sight in a desk hutch, drawer, and medicine cabinet. They stated that they did not like to see the bottles because it reminded them of cancer or they just did not like seeing the bottles.

#### Medication Organization and Pillboxes

In addition to having a specific location, 4 (20%, 4/20) patients had a system for organizing the medications that they found facilitated their adherence. For example, a 60-year-old married African American male with MM for 60 months kept his morning medications in his desk hutch on the right side, nighttime medications on the left, and cancer medications in the center. Placing the bottles in specific locations within the same drawer helped him remember when to take which medicines. A similar approach was used by a married Latino female with MM for 2 months who put all of her morning medications on 1 side of the bed and evening medications on the other side without special consideration of the MM medications.

The most sophisticated system was reported by a 67-year-old married white male with MM for 20 months. He kept a written diary with his medications in the original bottles next to his bed on a bed stand. He wrote down the time each dose of medications was taken. If he did not have time to use the diary, he took the medication and flipped the bottle upside down. When he returned home, he filled in the diary with an approximate time and flipped the bottle upright. Finally, a 40-year-old single African American with MM for 10 months stored all of her bottles in a special pouch that she found particularly attractive. The *cuteness* of the pouch was a source of pleasure contrasted with her feelings toward the contents of the pouch. She found it easy to locate the pouch in her bedroom and placed it in her purse when she went out.

A total of 6 (30%, 6/20) patients mentioned using pillboxes to hold their medications and 5 of those perceived this as a facilitator to taking their medications. However, this was not unanimous as a 50-year-old married African American male with MM for 50 months found the pillbox contributed to him confusing his morning and evening pills. He no longer used one, opting to keep the medicines in their original bottles.

#### Reminders

A total of 11 (55%, 11/20) patients described using specific visual and auditory reminders to take medications. Moreover, 7 patients commented that the location of their medicines served as a visual reminder to take the medicines, and the remaining 4 patients discussed auditory cell phone reminders. Of those, 2 used their cell phone alarm for evening medications and 2 used their cell phone calendar alert to remind them when to take their intermittent cancer medication.

#### Social Support

Social support from family was seen as an important facilitator of medication adherence for 10 (50%, 10/20) patients. A total of 3 different types of support were noted in the coding: medication reminders, emotional support to cope, and attendance at clinic visits to accurately capture information. Most commonly, patients described that a spouse provided a verbal reminder to take medications, which was described as wanted or helpful. One of the younger patients, a 33-year-old engaged African American female with MM for 61 months described relying heavily on her family for emotional support to cope along with tangible support to take her medication. Her son, in particular, often woke her up at night to remind her to take her evening medications. The 67-year-old married white male who used a written diary to track his medications described his wife and sister as being helpful at visits with the physician because they took written notes and reviewed them with him at home after each visit to make sure he understood exactly what medications to take and when to take them. Finally, it was noted that a 67-year-old married white female with MM for 17 months described that she and her husband “took care of each other” because he was also ill and in poor health. He reminded her to take her medications on occasions and also provided her with emotional support in her fight against MM.

#### Spirituality

A total of 3 patients discussed the importance of spirituality and God in their coping with medication adherence. For example, a 47-year-old married Latino female with MM for 2 months identified her spirituality as an important facilitator for adherence to her cancer medications. Despite having pre-existing diabetes, she described that the cancer medications were *overwhelming*. She believed in *divine healing* and “prayed for the cancer medications to heal her without causing side effects.” From her view, this worked as she had not experienced any side effects. As a result, she has been able “to cope with taking the cancer medications.”

### Perspectives on Sensored Medication Devices

Patients initial reactions to being presented with the SMDs were split with half reacting positively. Interestingly, several of the same SMD features were viewed positively by some and negatively by others. For example, participants were informed of the reminder features of SMDs such as text messaging and audio or visual alerts. A total of 2 patients were excited about the reminder alerts and 1 acknowledged that the alerts would be beneficial as she was already using her cell phone for this function; however, 3 (15%, 3/20) patients felt the alerts would be annoying, as expressed in other similar ways that can be summarized as “I don’t want that bottle talking to me/beeping at me.” Similarly, 4 (20%, 4/20) patients perceived they would benefit by having the provider notified of any missed doses. They liked the idea of having their providers gain access to the SMD data to “help monitor” them. Conversely, 3 (15%, 3/20) African American patients reacted strongly to the idea of their provider having access to the SMD information, seeing this as an invasion of privacy or “going too far.” Furthermore, they felt that it suggested the provider did not trust what the patient told them. As expressed by 1 patient, “I know that I am taking my medications and that is enough.”

The 60-year-old African American female who resided in a shelter and was open about her poor medication adherence felt that the SMDs would help her look forward to taking her medications. She found the technology novel and exciting. Moreover, 3 people mentioned that they liked the fact that they could “see what was going on,” referring to the Web-based platform that plotted the day and time that the bottle was open.

In addition, 3 patients were very satisfied with their current approach to medication management and simply did not like the idea that using an SMD required them to change their system. Moreover, 5 patients were not interested in using an SMD because they traveled or were out of the house often. They did not want to carry the bottle with all the pills when they only needed a few doses. They were concerned that if they removed all of the pills that they needed during their travels and left the bottle at home, this would be recorded inaccurately. Although seeing no personal benefit to using an SMD, these patients recognized the potential benefit to individuals who were older, had dementia, lived alone, or were otherwise struggling to remember to take their medications.

Several additional concerns were expressed even by those who were enthusiastic about the SMDs. Not surprisingly, privacy issues were identified by several patients. Some felt using the devices would be invasive even if used for a *good reason*, and a 67-year-old white married male who described himself as being comfortable with technology expressed that the SMDs made “a simple task too complicated.” He highlighted that some older people, not himself, would find the SMDs “too high tech.” Another pointed out that “many older people don’t have their cell phones with them all the time,” making the text reminders ineffective. Others focused on the bottle closure, suggesting that the lids would be difficult for people with arthritis to open or that they lids were not *childproof*. Furthermore, 2 patients were concerned that the cost of medications might go up if patients were asked to use SMDs. Finally, the 68-year-old African American married male stated that he was willing to use an SMD but was very clear that it was not foolproof. He expounded upon how someone could take out medication and never actually ingest it. As a result, he felt the technology was fatally flawed.

Although virtually everyone had a concern or doubt about the SMDs, when asked if they would be interested in trying a bottle in the future, 12 (60%, 12/20) patients expressed a willingness to test one out.

## Discussion

### Main Findings

The results of this qualitative study provide valuable insights into the medication-related attitudes of patients with MM and comorbid chronic conditions. With a median of 10 different medications per day, adherence to OAC medications was at times a challenge for 70% (14/20) of the patients. The inclusion of 80% (16/20) of patients who identified as African American further distinguishes the study. Attitudes toward SMDs identified concerns that could limit the willingness of some to engage with the technology.

### Barriers and Facilitators

Patients’ reported that barriers and facilitators of adherence provide rich data to inform intervention development. Of the 10 barriers to adherence identified, 7 are well known in the context of cancer: side effects, distractions, insurance or pharmacy delays, number of medications, pill size, fatigue, and spirituality [4,5]. In particular, 3 barriers have received less attention in the context of cancer. These included travel or being away from home, stigma, and homelessness. Cancer medications have historically been administered in the hospital setting. Treatment with OAC medications places new demands on the patient to manage their medications. Managing medications when patients are away from home is complicated by requirements for the safe handling of teratogenic OAC medications that are used to treat MM, such as thalidomide. As these precautions preclude removing medications from their original packaging, patients cannot simply take the doses they need. As reflected in our study, patients may choose to take their medications when they return home, which can contribute to missed doses and timing irregularities for subsequent doses.

Research on the stigma associated with cancer has focused largely on experiences of distress or impaired quality of life as opposed to medication adherence [24]. Moreover, 2 male African Americans mentioned concerns related to stigma, which impacted their adherence. Both described life experiences involving exposure to illicit drugs that affected their medication adherence—experiences not typically represented in cancer research.

Homelessness as a barrier to adherence is not novel. However, it is rare to have the voice of a homeless person undergoing cancer treatment represented in research. As revealed in her interview, adherence was challenged by the lack of routine and permanent location for her medications to be stored. However, she expressed feeling comfortable with technology, owned a smartphone, and embraced the potential of trying an SMD.

Patients’ adherence was facilitated by having a special location for medications, identifying an organizational structure, setting up visual or auditory reminders, receiving social support, and spirituality. Of those, medication organization, social support, and spirituality have received less attention in the context of medication adherence. There was a strong sense of ownership as patients discussed their management system, which was often informed by trial and error. Most notably, pillboxes worked for some but were abandoned by others who were confused (ie, mixed up morning and night medications) or burdened by them (ie, need to fill the boxes every week). Although identifying a consistent location and organizational system for medications may seem an obvious facilitator to adherence, we struggled to find published scientific articles at this granular level. Perhaps, this is so basic to clinical practice or pervasive among patients, it is not worthy of mention. However, patients’ attachment to their current system for storing and organizing their medications diminished interest in adopting SMDs for some in our sample and was recently noted as a barrier to adopting health-related technology [25].

The positive effect of social support on medication adherence has been reported in several studies across chronic disease states but significantly less so in cancer [26-28]. Despite this fact, half of the sample mentioned some aspect of social support as helpful in adhering to their medications. Spouses and children provided instrumental support with verbal reminders for or actually awaking patients to take their medications. Emotional support reinforced the willingness to take medications when patients were experiencing fatigue or feeling overwhelmed. Finally, information and instrumental support at provider appointments were important for capturing accurate information on treatment or medication. It is important to remember that simply living with family does not equate to having access to social support. A total of 2 patients resided with a spouse or family but perceived no outside support or assistance for their medication adherence. Finally, spirituality was mentioned by 3 patients as playing an important role in their adherence to OAC, which appears to have been a focus in only 1 prior publication among cancer patients [29].

Moreover, 60% (12/20) of the patients expressed a willingness to try an SMD, despite concerns. Most of the concerns related to either lack of privacy or were specific to elderly populations, such as bottles that were easy to open or comfort with technology. Privacy concerns are a common and expected barrier to the uptake of health-related technology [25]. Interestingly, patients differed in their response to the idea of their providers’ having access to their adherence data. This seemed to cross a line of trust for some African American patients in particular. A few patients identified initiation of their MM treatment as a time when they struggled with adherence because they felt confused and overwhelmed with all the medications. This suggests that the need for adherence support may vary over time and even some highly motivated patients might struggle with unintentional nonadherence, particularly at initiation of treatment.

### Study Limitations

Generalizability of the research findings is limited due to several factors. First, given the exploratory nature of the study, we recruited a small convenience sample of MM patients from 1 minority-serving academic institution in the Midwest United States. All the patients were established with their oncology providers, although they varied significantly in how long they had been diagnosed, which may have an impact on their experiences. Financial barriers to expensive anticancer medications were not an issue for any patient because the institution pursued avenues available to low-income individuals to access medications. As a result, financial barriers to adherence were limited to insurance co-payments, and this is uncommon in most settings [30]. Although 70% (14/20) of the patients admitted to missing doses of their cancer medications, the exact level of adherence was not captured or relevant to this study. It is also important to note that maximum adherence is required to gain optimal treatment effect with OAC medications. Regardless, the results are consistent with the larger cancer literature on adherence to OAC medications [4,5]. Finally, patients were not allowed to use an SMD before sharing their attitudes toward the tools. Considering that attitudes are often poor predictors of actual behaviors, no clear conclusions can be drawn about the potential for uptake of SMDs in this population. Nonetheless, SMDs are unlikely to be acceptable to all patients. Despite these limitations, to our knowledge, this is the first study to explore OAC medication adherence within patients from lower socioeconomic backgrounds and ethnic minorities who are often the most likely to struggle with adherence.

### Conclusions

Overall, the results of this small exploratory study in patients with MM are consistent with a growing body of research, suggesting that missed doses of OAC medication are common in cancer patients [31]. Advancing science in OAC medication adherence will require development and testing of theoretical models and not lists of barriers or facilitators as provided in this pilot. Novel interventions targeting adherence to OAC agents are beginning to emerge, and technology will likely have a role. These efforts need to include consideration of adherence to all prescribed medications and not exclusively OAC medications. SMDs can play a role in this research; however, patient concerns must be addressed. The knowledge gained from this exploratory study offers encouragement that an individual from lower socioeconomic backgrounds and ethnic minorities will be interested in being included in these efforts.

## Appendix

Multimedia Appendix 1 Interview guide.

## Abbreviations

MM: multiple myeloma
OAC: oral anticancer
SMD: sensored medication device

## References

[1] Weingart S, Brown E, Bach P, Eng K, Johnson S, Kuzel T, Langbaum T, Leedy R, Muller R, Newcomer L, O'Brien S, Reinke K, Rubino M, Saltz L, Walters R (2008). NCCN Task Force Report: oral chemotherapy. J Natl Compr Canc Netw.

[2] O'Neill VJ, Twelves CJ (2002). Oral cancer treatment: developments in chemotherapy and beyond. Br J Cancer.

[3] Bestvina CM, Zullig LL, Rushing C, Chino F, Samsa GP, Altomare I, Tulsky J, Ubel P, Schrag D, Nicolla J, Abernethy AP, Peppercorn J, Zafar SY (2014). Patient-oncologist cost communication, financial distress, and medication adherence. J Oncol Pract.

[4] Bassan F, Peter F, Houbre B, Brennstuhl MJ, Costantini M, Speyer E, Tarquinio C (2014). Adherence to oral antineoplastic agents by cancer patients: definition and literature review. Eur J Cancer Care (Engl).

[5] Puts M, Tu H, Tourangeau A, Howell D, Fitch M, Springall E, Alibhai S (2014). Factors influencing adherence to cancer treatment in older adults with cancer: a systematic review. Ann Oncol.

[6] Barillet M, Prevost V, Joly F, Clarisse B (2015). Oral antineoplastic agents: how do we care about adherence?. Br J Clin Pharmacol.

[7] Kumar SK, Dispenzieri A, Lacy MQ, Gertz MA, Buadi FK, Pandey S, Kapoor P, Dingli D, Hayman SR, Leung N, Lust J, McCurdy A, Russell SJ, Zeldenrust SR, Kyle RA, Rajkumar SV (2014). Continued improvement in survival in multiple myeloma: changes in early mortality and outcomes in older patients. Leukemia.

[8] Kumar SK, Rajkumar SV, Dispenzieri A, Lacy MQ, Hayman SR, Buadi FK, Zeldenrust SR, Dingli D, Russell SJ, Lust JA, Greipp PR, Kyle RA, Gertz MA (2008). Improved survival in multiple myeloma and the impact of novel therapies. Blood.

[9] Singhal S, Mehta J, Desikan R, Ayers D, Roberson P, Eddlemon P, Munshi N, Anaissie E, Wilson C, Dhodapkar M, Zeddis J, Barlogie B (1999). Antitumor activity of thalidomide in refractory multiple myeloma. N Engl J Med.

[10] Rajkumar S, Jacobus S, Callander NS, Fonseca R, Vesole DH, Williams ME, Abonour R, Siegel DS, Katz M, Greipp PR, Eastern Cooperative Oncology Group (2010). Lenalidomide plus high-dose dexamethasone versus lenalidomide plus low-dose dexamethasone as initial therapy for newly diagnosed multiple myeloma: an open-label randomised controlled trial. Lancet Oncol.

[11] Lacy MQ, Allred JB, Gertz MA, Hayman SR, Short KD, Buadi F, Dispenzieri A, Kumar S, Greipp PR, Lust JA, Russell SJ, Dingli D, Zeldenrust S, Fonseca R, Bergsagel PL, Roy V, Stewart AK, Laumann K, Mandrekar SJ, Reeder C, Rajkumar SV, Mikhael JR (2011). Pomalidomide plus low-dose dexamethasone in myeloma refractory to both bortezomib and lenalidomide: comparison of 2 dosing strategies in dual-refractory disease. Blood.

[12] Moreau P, Masszi T, Grzasko N, Bahlis NJ, Hansson M, Pour L, Sandhu I, Ganly P, Baker BW, Jackson SR, Stoppa A, Simpson DR, Gimsing P, Palumbo A, Garderet L, Cavo M, Kumar S, Touzeau C, Buadi FK, Laubach JP, Berg DT, Lin J, Di BA, Hui A, van de Velde H, Richardson PG, TOURMALINE-MM1 Study Group (2016). Oral ixazomib, lenalidomide, and dexamethasone for multiple myeloma. N Engl J Med.

[13] San-Miguel J, Hungria V, Yoon S, Beksac M, Dimopoulos M, Elghandour A, Jedrzejczak W, G&uuml;nther A, Nakorn T, Siritanaratkul N, Corradini P, Chuncharunee S, Lee J, Schlossman R, Shelekhova T, Yong K, Tan D, Numbenjapon T, Cavenagh J, Hou J, LeBlanc R, Nahi H, Qiu L, Salwender H, Pulini S, Moreau P, Warzocha K, White D, Blad&eacute; J, Chen W, de la Rubia J, Gimsing P, Lonial S, Kaufman J, Ocio E, Veskovski L, Sohn S, Wang M, Lee J, Einsele H, Sopala M, Corrado C, Bengoudifa B, Binlich F, Richardson P (2014). Panobinostat plus bortezomib and dexamethasone versus placebo plus bortezomib and dexamethasone in patients with relapsed or relapsed and refractory multiple myeloma: a multicentre, randomised, double-blind phase 3 trial. Lancet Oncol.

[14] Kumar SK, Callander NS, Alsina M, Atanackovic D, Biermann JS, Chandler JC, Costello C, Faiman M, Fung HC, Gasparetto C, Godby K, Hofmeister C, Holmberg L, Holstein S, Huff CA, Kassim A, Liedtke M, Martin T, Omel J, Raje N, Reu FJ, Singhal S, Somlo G, Stockerl-Goldstein K, Treon SP, Weber D, Yahalom J, Shead DA, Kumar R (2017). Multiple myeloma, version 3.2017, NCCN Clinical Practice Guidelines in oncology. J Natl Compr Canc Netw.

[15] Huntington S, Weiss B, Vogl D, Cohen A, Garfall A, Mangan P, Doshi J, Stadtmauer E (2015). Financial toxicity in insured patients with multiple myeloma: a cross-sectional pilot study. Lancet Haematol.

[16] MacEwan JP, Batt K, Yin W, Peneva D, Sison S, Vine S, Chen C (2018). Economic burden of multiple myeloma among patients in successive lines of therapy in the United States. Leuk Lymphoma.

[17] Costa LJ, Brill IK, Omel J, Godby K, Kumar SK, Brown EE (2017). Recent trends in multiple myeloma incidence and survival by age, race, and ethnicity in the United States. Blood Adv.

[18] Kavookjian J, Wittayanukorn S (2015). Interventions for adherence with oral chemotherapy in hematological malignancies: a systematic review. Res Social Adm Pharm.

[19] Silverman J, Krieger J, Kiefer M, Hebert P, Robinson J, Nelson K (2015). The relationship between food insecurity and depression, diabetes distress and medication adherence among low-income patients with poorly-controlled diabetes. J Gen Intern Med.

[20] Burhenn PS, Smudde J (2015). Using tools and technology to promote education and adherence to oral agents for cancer. Clin J Oncol Nurs.

[21] Kyle RA, Rajkumar SV (2007). Epidemiology of the plasma-cell disorders. Best Pract Res Clin Haematol.

[22] Gale N, Heath G, Cameron E, Rashid S, Redwood S (2013). Using the framework method for the analysis of qualitative data in multi-disciplinary health research. BMC Med Res Methodol.

[23] Miceli TS, Colson K, Faiman BM, Miller K, Tariman JD, International Myeloma Foundation Nurse Leadership Board (2011). Maintaining bone health in patients with multiple myeloma: survivorship care plan of the International Myeloma Foundation Nurse Leadership Board. Clin J Oncol Nurs.

[24] Chambers SK, Baade P, Youl P, Aitken J, Occhipinti S, Vinod S, Valery PC, Garvey G, Fong KM, Ball D, Zorbas H, Dunn J, O'Connell DL (2015). Psychological distress and quality of life in lung cancer: the role of health-related stigma, illness appraisals and social constraints. Psychooncology.

[25] Simblett S, Greer B, Matcham F, Curtis H, Polhemus A, Ferrão J, Gamble P, Wykes T (2018). Barriers to and facilitators of engagement with remote measurement technology for managing health: systematic review and content analysis of findings. J Med Internet Res.

[26] DiMatteo M (2004). Social support and patient adherence to medical treatment: a meta-analysis. Health Psychol.

[27] Chlebowski RT, Kim J, Haque R (2014). Adherence to endocrine therapy in breast cancer adjuvant and prevention settings. Cancer Prev Res (Phila).

[28] Magrin M, D'Addario M, Greco A, Miglioretti M, Sarini M, Scrignaro M, Steca P, Vecchio L, Crocetti E (2015). Social support and adherence to treatment in hypertensive patients: a meta-analysis. Ann Behav Med.

[29] Hefner J, Csef E, Kunzmann V (2017). Adherence and coping strategies in outpatients with chronic myeloid leukemia receiving oral tyrosine kinase inhibitors. Oncol Nurs Forum.

[30] Niccolai J, Roman D, Julius J, Nadour R (2017). Potential obstacles in the acquisition of oral anticancer medications. J Oncol Pract.

[31] Greer JA, Amoyal N, Nisotel L, Fishbein JN, MacDonald J, Stagl J, Lennes I, Temel JS, Safren SA, Pirl WF (2016). A systematic review of adherence to oral antineoplastic therapies. Oncologist.

