# Supplementary material for: Barriers and Facilitators of Using Sensored Medication Adherence Devices in a Diverse Sample of Patients With Multiple Myeloma: Qualitative Study
Source: JMIR Cancer. 2018 Nov 12;4(2):e12. doi: 10.2196/cancer.9918 (PMC6256103; doi:10.2196/cancer.9918)
Supplement: Multimedia Appendix 1 [file cancer_v4i2e12_app1.pdf]

Appendix 1. Interview Guide

1. I want to begin by asking a few questions to get a sense of your use of technology. What type of technology, if any, do you use?
2. Do you own a cellphone? Probe: use of internet, texting, & cellphone applications.
3. Next I am going to focus more on your health. How was your health before you were diagnosed with multiple myeloma? Probe: experience with taking daily medications
4. What other chronic diseases do you have now aside from the cancer? Probe: experience with taking daily medications
5. Which of the medications you are taking these days do you feel are the most important? Why is that?
6. I am curious to know how you organize or manage those medications. Probe: use of pillbox
7. How do you remember to take your medications at home? Probe: anything else? Probe: if they have not mentioned, what type of help do you get with your medications at home?
8. Do you ever forget to take your cancer medications? If yes, what things are going on when that happens?
9. What other things make it hard for you to take your medications?
